# Studies on ageing and the severity of radiographic joint damage in rheumatoid arthritis

**DOI:** 10.1186/s13075-015-0740-0

**Published:** 2015-08-24

**Authors:** Lukas Mangnus, Hanna W. van Steenbergen, Elisabet Lindqvist, Elisabeth Brouwer, Monique Reijnierse, Tom WJ Huizinga, Peter K. Gregersen, Ewa Berglin, Solbritt Rantapää-Dahlqvist, Désirée van der Heijde, Annette HM van der Helm-van Mil

**Affiliations:** Department of Rheumatology, Leiden University Medical Center, P.O. Box 9600, 2300 RC Leiden, The Netherlands; Department of Rheumatology, Lund University and Skåne University hospital Lund, Lund, Sweden; Department of Rheumatology, University Medical Center Groningen, Groningen, Netherlands; Department of Radiology, Leiden University Medical Center, Leiden, Netherlands; Feinstein Institute for Medical Research and North Shore–LIJ Health System, Manhasset, New York USA; Department of Public Health and Clinical Medicine/Rheumatology, University Hospital, Umea, Sweden

## Abstract

**Introduction:**

The western population is ageing. It is unknown whether age at diagnosis affects the severity of Rheumatoid Arthritis (RA), we therefore performed the present study.

**Method:**

1,875 RA-patients (7,219 radiographs) included in five European and North-American cohorts (Leiden-EAC, Wichita, Umeå, Groningen and Lund) were studied on associations between age at diagnosis and joint damage severity. In 698 Leiden RA-patients with 7-years follow-up it was explored if symptom duration, anti-citrullinated-peptide-antibodies (ACPA), swollen joint count (SJC) and C-reactive-protein (CRP) mediated the association of age with joint damage. Fifty-six other RA-patients of the EAC-cohort underwent baseline MRIs of wrist, MCP and MTP-joints; MRI-inflammation (RAMRIS-synovitis plus bone marrow edema) was also evaluated in mediation analyses. Linear regression and multivariate normal regression models were used.

**Results:**

Analysis on the five cohorts and the Leiden-EAC separately revealed 1.026-fold and 1.034-fold increase of radiographic joint damage per year increase in age (β=1.026, 1.034, both p<0.001); this effect was present at baseline and persisted over time. Age correlated stronger with baseline erosion-scores compared to joint space narrowing (JSN)-scores (r=0.38 versus 0.29). Symptom duration, ACPA, SJC and CRP did not mediate the association of age with joint damage severity. Age was significantly associated with the MRI-inflammation-score after adjusting for CRP and SJC (β=1.018, p=0.027). The association of age with joint damage (β=1.032, p=0.004) decreased after also including the MRI-inflammation-score (β=1.025, p=0.021), suggesting partial mediation.

**Conclusion:**

RA-patients presenting at higher age have more severe joint damage; this might be partially explained by more severe MRI-detected inflammation at higher age.

**Electronic supplementary material:**

The online version of this article (doi:10.1186/s13075-015-0740-0) contains supplementary material, which is available to authorized users.

## Introduction

The western population is ageing. Consequently, the number of patients with rheumatoid arthritis (RA) presenting at an older age is increasing [1, 2]. Ageing is associated with alterations and remodelling of the innate and adaptive immune system (immunosenescence) [3–5]. It is unclear to what extent ageing or age-associated changes in function of immune cells influence the severity of RA. If RA severity differs for patients diagnosed at different age categories, this is relevant for clinical practice.

Some previous studies suggest that older patients with RA have more joint damage [6–11], whereas other studies observed no difference [12, 13] or observed less joint damage in older patients with RA [14]. Most studies performed analyses at a single time point [7–10, 13, 14] and all studied patients categorized as younger or older [6–14]. The first aim of the present study was to explore the association between age and severity of joint damage in more detail. Patients with RA included in one North-American and four European longitudinal cohorts were studied for severity of joint damage at disease presentation and during the course of the disease. We analysed age as a continuous variable to obtain optimal insight into the effects of age.

Second, no studies have explored processes underlying the association between age at disease onset and radiographic joint damage. First, because joint damage measures such as the Sharp-van der Heijde (SHS)-score assess bone erosions and joint space narrowing (JSN), JSN may occur not only due to RA but also reflect degeneration. An increase in total SHS severity at older age could therefore be due to a disproportional increase in JSN. Additionally, based on general knowledge of risk factors for progressive joint damage in RA (longer symptom duration, presence of RA-related auto-antibodies, higher numbers of swollen joints and elevated acute-phase reactants are all associated with more severe damage), we made several other hypotheses. We assumed that older patients present at a later point in time, and therefore have more severe joint damage. In addition, as the prevalence of RA-related auto-antibodies in the general population increases with increasing age, we hypothesized that patients with RA presenting at older age are more often rheumatoid factor (RF)-positive or anti-citrullinated peptide antibodies (ACPA)-positive and therefore have more severe disease [8, 11–13, 15–20]. Likewise we postulated that inflammation at diagnosis is more severe at older age resulting in more joint damage. Inflammation was evaluated using traditional measures (swollen joint count (SJC), C-reactive protein (CRP)) and using magnetic resonance imaging (MRI), which is more sensitive in detecting local inflammation [21, 22]. We also aimed to explore these hypotheses.

Thus the first aim of this study was to explore the association of age with joint damage severity in more detail and, second, we aimed to increase the understanding of the processes underlying the association between age of disease onset and the severity of the disease course.

## Methods

### Study population

To determine the association between age at diagnosis and severity of joint damage, patients with RA included in five longitudinal inception cohorts were studied. In total this comprised 1,875 patients with 7,219 sets of radiographs made at baseline and during follow up. Patients were included in cohorts of the Leiden early arthritis clinic (EAC), Groningen (both the Netherlands), Wichita (USA), Umeå and Lund (both Sweden). All patients with RA fulfilled the 1987 criteria for RA except for the Lund cohort where the 1958 criteria were used. The age at diagnosis was recorded in all cohorts. For all studies the regional ethics committee approved the study and all participants gave their written informed consent. Extensive descriptions of these cohorts are presented elsewhere [23–26], short descriptions are provided below.

#### Leiden early arthritis clinic (EAC) cohort

Patients with early RA (n = 698) between 1993 and 2006 were included [23]. From these patients 3,643 sets of radiographs of the hands and feet were obtained during 7 years of follow up. Follow-up visits, including radiographs, were done yearly. All radiographs were chronologically scored by an experienced reader, blinded to the clinical data, according to the SHS-method. The intra-reader intra-class correlation coefficient (ICC) was 0.91. The applied treatment strategies changed over time, as described elsewhere [27]; the inclusion periods were used to adjust for differences in applied treatments. The data for these patients were used for the mediation analyses as they contained most radiographs and extensive data on clinical and serologic variables.

A second study population of patients with RA was included in the EAC in 2010–2012. In addition to the general EAC-protocol including radiographs, these consecutively included patients had contrast-enhanced 1.5 T MRI of unilateral metacarpophalangeal (MCP), wrist and metatarsophalangeal (MTP) joints at baseline. Patients who had 1-year follow up, including radiographs, were selected (n = 56). The MRI (ONI-MSK-Extreme 1.5 T MRI (GE, WI, USA)) was performed at inclusion, on the most symptomatic side or the dominant side in the case of equally severe symptoms. Scanning was done according to the RA MRI score (RAMRIS), with contrast enhancement. The scan protocol is described in Additional file 1. MRI-scoring was done by two trained readers blinded to any clinical data [28–30]. The within-reader ICCs were 0.98 and 0.83, and the inter-reader ICC 0.82. The mean score of both readers was studied.

#### Wichita

This North-American cohort consisted of 293 patients that were diagnosed between 1963 and 1999 [24]. In this cohort 1,062 radiographs were made during 15 years of follow up. All radiographs were chronologically scored by an experienced reader using the SHS (ICC 0.98) [31].

#### Umeå

The third cohort consisted of 459 patients included between 1995 and 2010 [6]. These patients had radiographs at baseline and 2 years: 868 radiographs were obtained and were scored by two trained rheumatologists according to the Larsen score [32, 33]. Treatment strategies differed between 1995 and 1999, 2000 and 2005, and 2006 and 2010, with less severe radiographic progression in the subsequent treatment periods.

#### Groningen

This dataset included 278 patients with RA who were diagnosed between 1945 and 2001. During 14 years of follow up 865 radiographs were obtained that were chronologically scored according to the SHS by one of two readers (intra-reader ICC >0.90 inter-reader ICC 0.96) [34]. Joint destruction was less severe after 1990, which coincides with the introduction of treatment with disease-modifying antirheumatic drugs (DMARDs).

#### Lund

This cohort consist of 147 patients recruited from primary care units in the area of Lund from 1985–1989 [26]: 781 radiographs were obtained during 5 years of follow up. These radiographs were scored according to the Larsen score (ICC 0.94) [32, 35]. All results for age in all datasets in this study represent the age of the patient at the time of diagnosis. At the baseline visit, the date of birth or age itself was recorded.

### Analyses

First, the associations between age at diagnosis and the severity of joint damage were evaluated for each cohort separately. A multivariate normal regression model for longitudinal data [27] was used with radiographic scores as the outcome and age as the continuous independent variable. The radiographic scores were log10-transformed (log10 (score+1)) to approximate a normal distribution. In all cohorts the residuals of the models were normally distributed around zero, indicating a good fit of the models (Additional file 2: Figure S1). This repeated measurement analysis takes the correlation between repeated measurements within patients into account. A heterogeneous first-order autoregressive (ARH1) correlation structure was used, assuming a stronger correlation for measurements taken in a shorter period of time than for those over a longer period. As described elsewhere in detail [27] this model is able to test for two effects: first the model can be used to analyse whether patients with a risk factor have more severe joint damage at any point in time; this reflects a constant effect size over time.

Second, the model can be used to analyse whether patients have more severe radiographic progression over time; this present the steepness of the curve of joint damage over time (risk factor*time). In the evaluated cohorts, the radiographic data were plotted (before starting with statistical analysis); this suggested an effect that is constant over time and not a progression effect. Therefore analyses were focussed on the constant effect and all results presented (effect sizes, *p* values) were those of a constant effect, thus, this concerns a difference in joint destruction that was equally present at every time point.

In all datasets, analyses were adjusted for gender. In the cohorts that included patients in periods with different treatments strategies (EAC, Umeå and Groningen) an additional adjustment was made for the inclusion period (as proxy for treatment strategy) as radiographic joint damage varied between different inclusion periods. As the analyses were performed on the log-scale, the resulting effect estimates were back-transformed to the normal scale and indicated the fold increase in joint damage per year of increase in age at diagnosis. Thus, this outcome indicates a relative increase in joint damage and is unit-free. This allowed us to enter the effects of the five different cohorts in an inverse-weighted meta-analysis. This method weights the results with a low standard error stronger than results with a higher standard error, preventing over-representation of the less precise data on the overall outcome. A random-effect model was used.

To explore whether the increase in total SHS at increasing age was explained by a disproportionate increase in JSN, possibly reflecting age-related degenerative changes, the total SHS at baseline of the 698 Leiden RA patients was split into erosion score and JSN score. Pearson’s correlation coefficients were determined and equality/inequality of the two correlation coefficients was assessed using the corcor command. Similarly, the total SHS was split for separate locations; predilection locations for primary osteoarthritis (proximal interphalangeal (PIP)-joints, carpometacarpal-1 (CMC-1)-joints and MTP-1 plus interphalangeal-1 joints were compared to other joints.

**Fig. 1:**
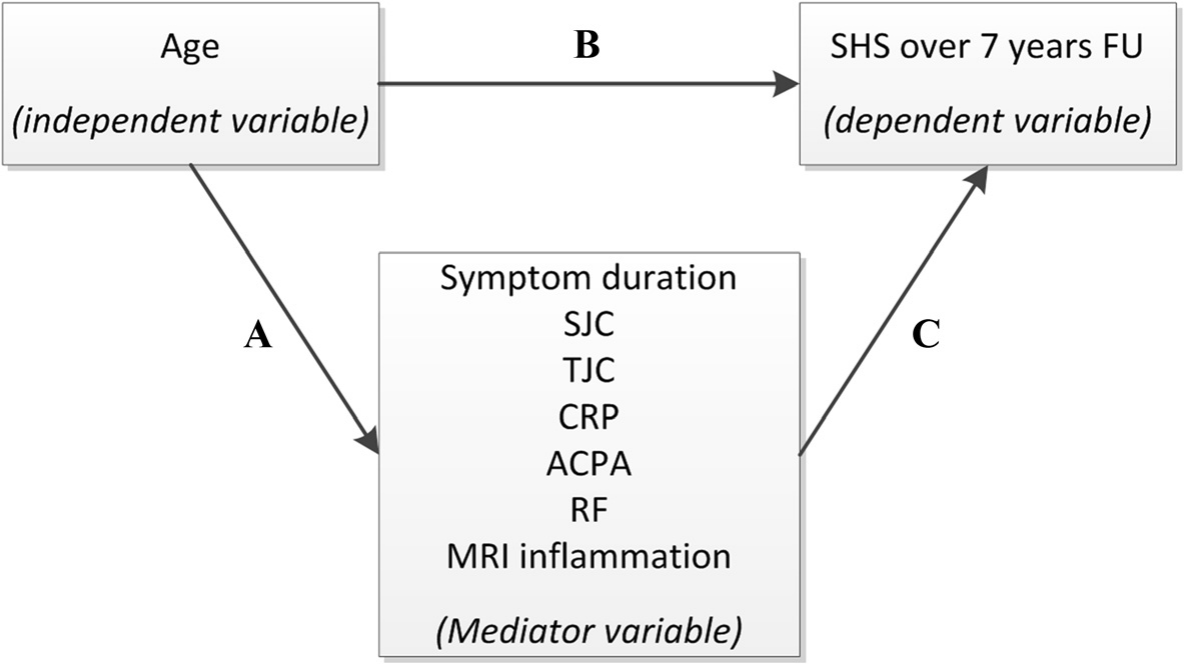
Schematic overview of the causal paths that were studied using mediation models as described by Baron and Kenny. The figure illustrates two causal paths that lead to an outcome. A direct path from independent to dependent variable (**b**) and an indirect path from an independent to a dependent variable through a mediator variable (**a,c**). To test mediation three tests have to be performed according to Baron and Kenny [36]. 1) The mediator variables were regressed on the independent variable (**a**), the independent variable should significantly affect the mediator variables. 2) regression analysis of the dependent variable on the independent variable was done (**b**); in this analysis the independent variable must significantly affect the dependent variable. 3) The dependent variable was regressed on the independent and mediator variable (**b** and **c**). When mediation occurs the mediator variable significantly affects the dependent variable and the effect of the independent variable on the dependent variable is closer to zero. In this study we tested whether different mediators could influence the effect of age on radiographic joint damage. The tested mediators were symptom duration at diagnosis, swollen joint count (*SJC*), tender joint count (*TJC*), C-reactive protein (*CRP*), anti-citrullinated protein antibody (*ACPA*), rheumatoid factor (*RF*), and inflammation detected on magnetic resonance imaging (*MRI*). *SHS* Sharp-van der Heijde score

Mediation analyses were used to identify potential mechanisms underlying the association between age at disease onset and radiographic joint damage. Mediation analyses were performed according to Baron and Kenny (Fig. 1) [36]. First (step 1), the mediator variables were regressed on the independent variable (age); the independent variable should significantly affect the mediator variables. Second (step 2), regression analysis of the dependent variable (radiographic joint damage) on the independent variable (age) was done. In this analysis the independent variable must significantly affect the dependent variable. Third (step 3), the dependent variable was regressed on the independent and mediator variable. When mediation occurs, the mediator variable significantly affects the dependent variable and the effect of the independent variable on the dependent variable decreases (partial mediation) or disappears completely (full mediation). Symptom duration at baseline, ACPA (anti-CCP2), IgM-RF, CRP, SJC and TJC were assessed as mediators. Linear or logistic regression was used as appropriate for step 1. For steps 2 and 3 the multivariate normal regression model was used as described.

Analyses were performed with SPSS V20.0.0; the meta-analysis and the equality of the correlation test were performed using STATA/SE V12.1.

## Results

### Age at diagnosis and severity of radiographic joint damage

**Fig. 2:**
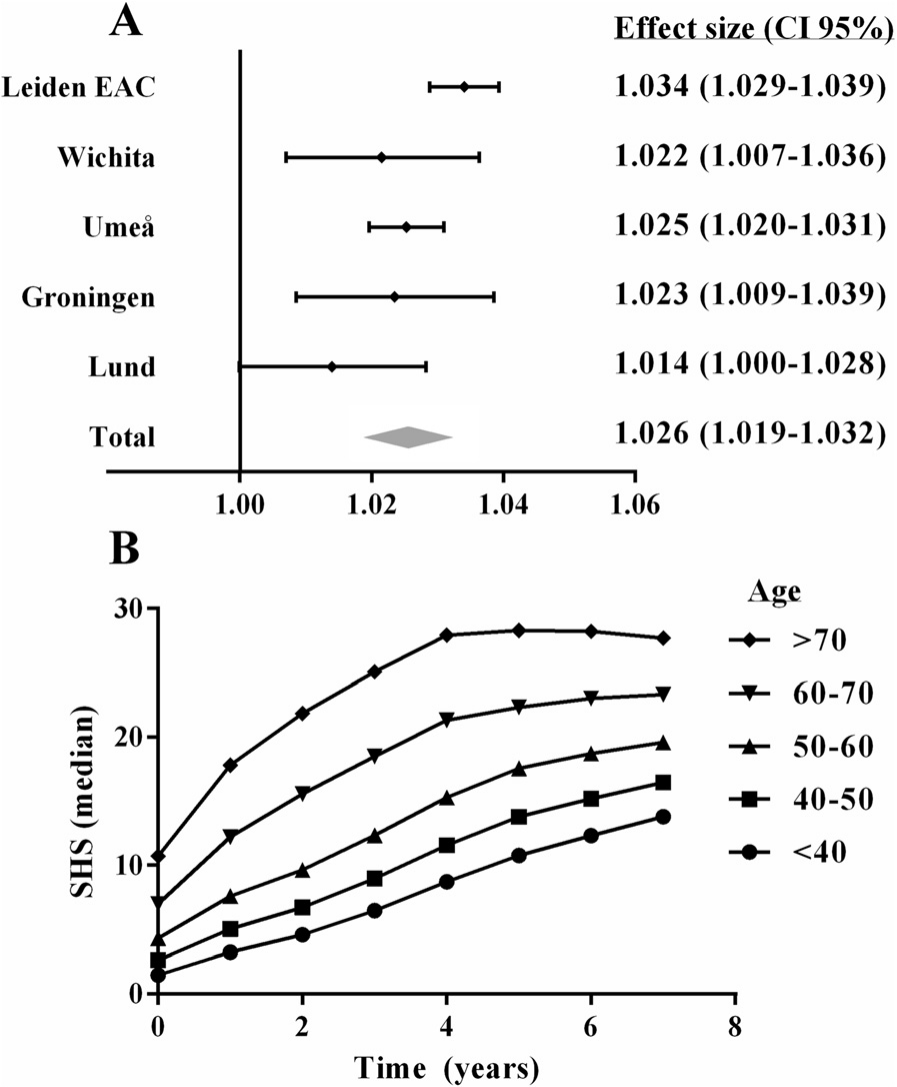
Association between age at diagnosis and severity of joint damage in five longitudinal cohorts summarized in a meta-analysis (**a**) and depicted for patients with rheumatoid arthritis included in the Leiden Early Arthritis Clinic (*EAC*) for different age categories (**b**). **a** Age was entered as a continuous variable in the multivariate normal regression analysis, because the plots of the raw data suggested no interaction of age with time. The meta-analysis (inverse weighted meta-analysis with a random-effect model) summarizes the effects of the age of the different cohorts. An effect size of 1.034 represents a 1.034-fold increase in joint damage per year increase in age. Because these effect sizes were unit-free they could be compared in meta-analysis. **b** Although age was analyzed as a continuous variable, the predicted Sharp-van der Heijde (*SHS*) scores per age-category were plotted to illustrate the data. The SHS scores predicted by the multivariate normal regression analysis are presented

First, the association between age and severity of joint damage observed on radiographs was explored separately in each cohort. Baseline characteristics of these patients are presented in Table 1. In all cohorts an increase in age at diagnosis was associated with more severe joint damage at baseline and this effect persisted over time. Combining all five cohorts in a meta-analysis revealed that patients had 1.026-fold more joint damage observed on radiographs per year increase in age at any point during the disease course (*β* = 1.026, *p* <0.001, Fig. 2a). For illustration, the predicted severity of joint damage per group of patients according to different age categories is depicted in Fig. 2b, based on 698 patients with RA included in the Leiden-EAC. Data for the other cohorts are depicted in Additional file 3: Figure S2. Here joint damage increased 1.034-fold per year increase in age; this effect was constant over time (*β* = 1.034, *p* <0.001).

**Table 1:** Characteristics of patients with rheumatoid arthritis included in the longitudinal cohorts studied. Age, symptom duration, TJC, SJC, CRP, ACPA and RF were assessed at baseline

	EAC Part 1	EAC Part 2 (MRI)	Wichita	Umeå	Groningen	Lund
Total number of patients	698	56	293	459	278	147
Total number of radiographs	3.643	105	1.062	868	865	781
Mean number of radiographs per patient (SD)	5.2 (2.1)	1.9 (0.3)	3.6 (2.0)	1.9 (0.3)	3.1 (1.4)	5.3 (0.8)
Year of diagnosis	1993–2006	2010–2012	1963–1999	1995–2010	1945–2001	1985–1989
Radiographic follow up in years	7	1	15	2	14	5
Method of scoring	SHS	SHS	SHS	Larsen	SHS	Larsen
Age, years						
Mean (SD)	56.6 (15.6)	55.9 (14.2)	48.8 (14.2)	53.9 (14.5)	49.3 (12.6)	50.7 (11.5)
Median (IQR)	58 (46–68)	59 (46–65)	49 (39–60)	56 (45–64)	50 (40–59)	51 (43–59)
Range	17.1–92.4	21.5–77.8	16.0–83.0	17.0–83.0	18.3–76.3	18.0–78.0
Female sex (%)	474 (67.8)	31 (55.4)	226 (77.1)	321 (69.2)	196 (70.5)	98 (66.7)
Symptom duration in weeks (IQR)	19 (11–37)	18 (11–32)	NA	NA	NA	43 (29–62)
TJC (IQR)	8 (5.0–12.0)	7 (4.0–10.5)	NA	NA	NA	NA
SJC (IQR)	8 (4.0–14.0)	5 (3.5–10.0)	NA	NA	NA	NA
ESR (IQR)	33 (19–54)	25 (10–41)	NA	NA	NA	NA
CRP (IQR)	17 (8.0–40.0)	11 (3.0–20.5)	NA	NA	NA	NA
ACPA positivity (%)	365 (53.7)	33 (63.5)	238 (82.1)	339 (73.1)	162 (79.4)	114 (80.3)
RF positivity (%)	405 (58.2)	36 (64.3)	NA	NA	259 (93.8)	115 (80.3)

Age, symptom duration, TJC, SJC, ESR, CRP, ACPA and RF were assessed at baseline

*EAC* Early Arthritis Clinic, *MRI* magnetic resonance imaging, *TJC* tender joint count, *SJC* swollen joint count, *CRP* C-reactive protein, ESR erythrocyte sedimentation rate, *ACPA* anti-citrullinated peptide antibodies, *RF* rheumatoid factor, *SHS* Sharp-van der Heijde

### Correlation of age with erosion score and JSN score

**Fig. 3:**
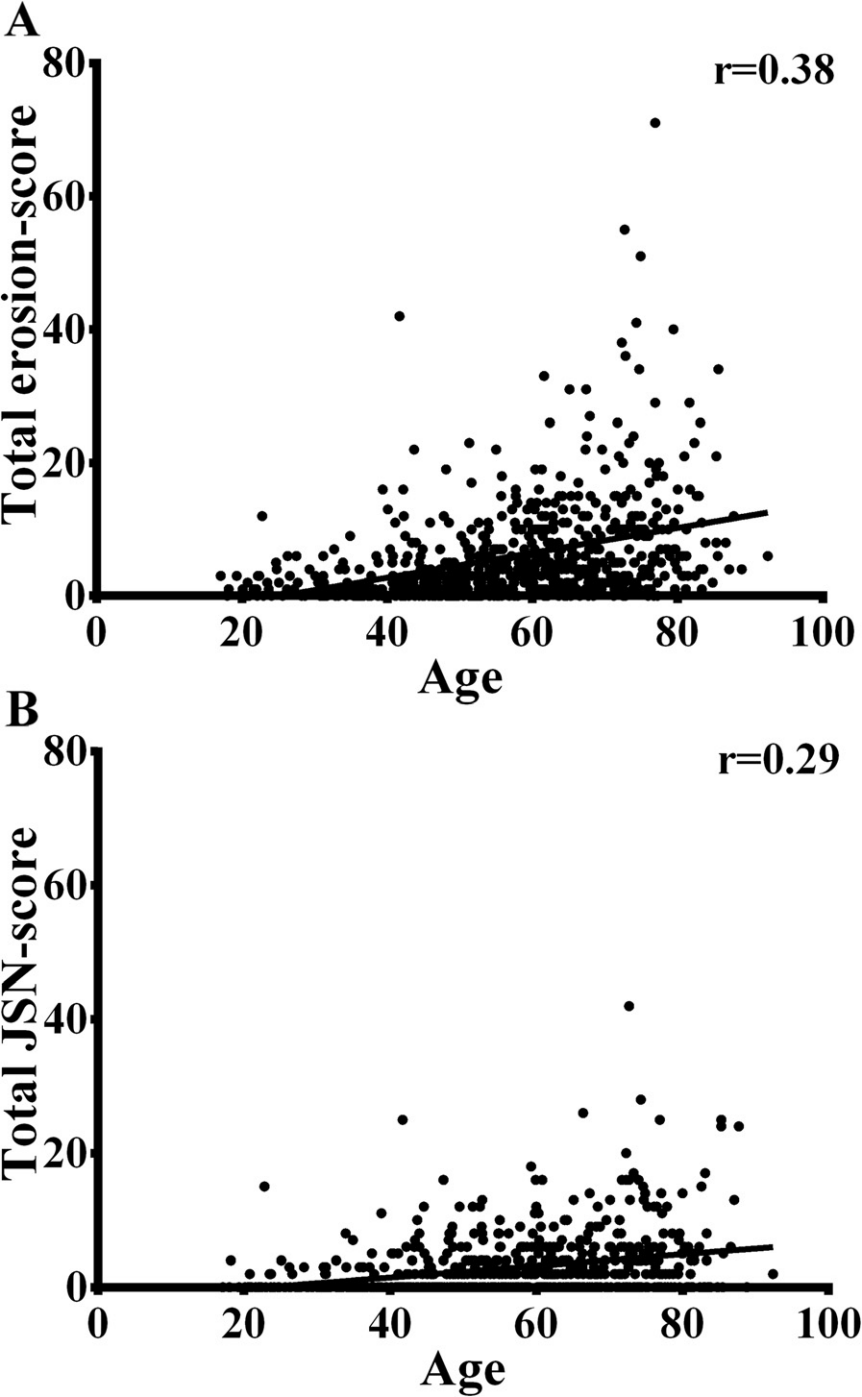
Correlation between age and Sharp-van der Heijde erosion (**a**) and joint space narrowing scores (*JSN*) (**b**) at baseline

**Fig. 4:**
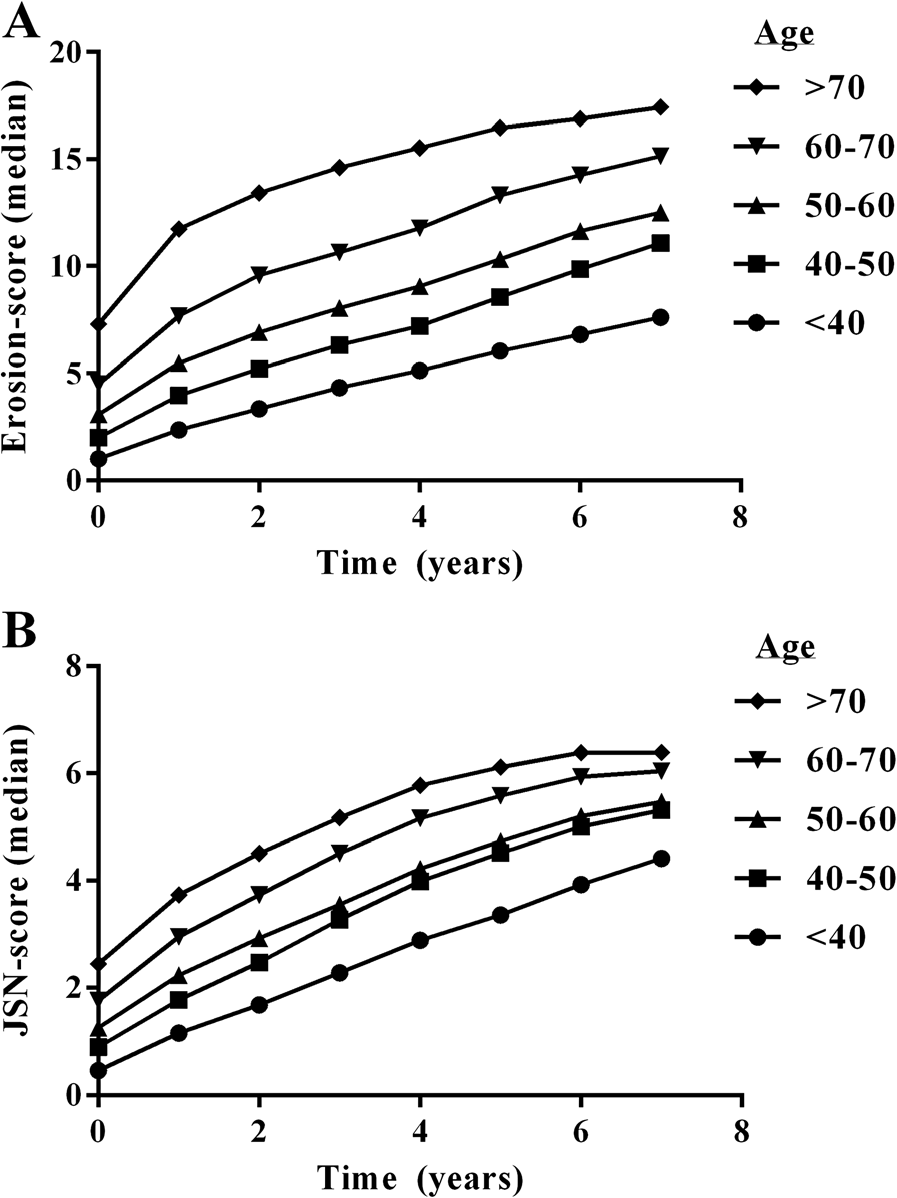
Sharp-van der Heijde erosion score (**a**) and joint space narrowing score (**b**) over time for patients with rheumatoid arthritis from the Early Arthritis Clinic, categorized by age at diagnosis

The SHS at disease onset in patients with RA included in the Leiden EAC were split into total erosion scores and total JSN scores to explore whether age-related degenerative changes explain the association between age and total SHS score, and we assessed whether the correlation between age and JSN score was stronger than the correlation between age and the erosion score. Age at diagnosis was significantly correlated with both the erosion and JSN scores (*r* = 0.38, *p* <0.001 and *r* = 0.29, *p* <0.001 respectively, Fig. 3) at baseline. The correlation coefficient of the erosion score was significantly higher than that of the JSN score (*p* = 0.006). The association between age and erosion and JSN score over time was also assessed, and older age was associated with higher erosion scores and higher JSN scores at all points in time (*β* = 1.030, *p* <0.001, and *β* = 1.020, *p* <0.001, respectively) (Fig. 4).

The total SHS score is also the sum of the scores of different joints. We hypothesized that some joints (predilection locations for primary osteoarthritis in particular, such as CMC-1) are more frequently affected by age-related degeneration. Therefore the total SHS score at baseline was split for several regions and correlations between the SHS score per region and age were determined. This revealed that the correlation of CMC-1 was comparable to that of the wrist (*r* = 0.26 and *r* = 0.23, both *p* <0.001). The correlation of the other locations was also comparable (*r* = 0.20, 0.31 and 0.39 for MTP + IP-1, MCP joints and PIP joints, all *p* <0.001), only in the MTP2−5 joints the correlation of the SHS score with age was lower (*r* = 0.08, *p* = 0.03, Additional file 4: Figure S3). Together these results suggest that degenerative changes at older age may contribute to more severe joint damage in patients presenting at older age but are clearly insufficient to explain the total effect.

### Age and symptom duration at first presentation

Because it is known that prolonged symptom duration at first presentation is associated with more severe radiographic progression [37, 38], we hypothesized that older persons present with a longer symptom duration, hence mediating the association with joint damage. Although longer symptom duration was associated with more joint damage (*β* = 1.003, *p* <0.001), symptom duration was inversely associated with age, (*β* = 0.99, *p* <0.001), hence older age was associated with shorter symptom duration. Therefore, symptom duration did not mediate the effect of age on joint damage (Table 2).

**Table 2:** Mediation analysis in 698 patients with rheumatoid arthritis from the Leiden Early Arthritis Clinic, with radiographic severity of joint damage over 7 years as the outcome variable

Step 1: effect of age on possible mediators			
	Effect (*β*)^a^	95 % CI	*p* value
SJC	1.00	1.00–1.01	0.11
TJC	1.00	1.00–1.01	0.55
Symptom duration	0.992	0.988–0.996	<0.001
CRP	1.016	1.011–1021	<0.001
RF	0.99	0.98–1.00	0.09
ACPA	0.98	0.97–0.99	<0.001
Step 2: effect of age on radiographic joint damage			
	Effect (*β*)	95 % CI	*p* value
Ageing	1.034	1.029–1.040	<0.001
Step 3: effect of age and possible mediator on radiographic joint damage			
	Effect (*β*)	95 % CI	*p* value
SJC	1.00	0.99–1.00	0.23
Ageing	1.035	1.029–1.040	<0.001
TJC	1.00	0.98–1.01	0.76
Ageing	1.037	1.030–1.044	<0.001
Symptom duration	1.003	1.002–1.005	<0.001
Ageing	1.035	1.029–1.040	<0.001
CRP	1.003	1.001–1.005	0.003
Ageing	1.033	1.027–1.038	<0.001
ACPA	1.37	1.16–1.60	<0.001
Ageing	1.035	1.030–1.040	<0.001
RF	1.30	1.10–1.52	0.002
Ageing	1.034	1.029–1.039	<0.001

^a^The effect size (*β*) of swollen joint count (SJC), tender joint count (TJC), symptom duration and C-reactive protein (CRP) reflect the increase per year increase of age. For example, the *β* for CRP is 1.016 this means that for every year increase in age there is 1.016-fold increase in CRP. A *β* of 0.992 indicates an increase 0.992- fold, hence actually a decrease. The effect size of anti-citrullinated protein antibody (ACPA) and rheumatoid factor (RF) reflect the odds ratio. Step 1, 2 and 3 of the mediation analyses are explained in Fig. 1. In step 1 a linear or logistic regression was used, in step 2 and 3 a multivariate normal regression analysis was used [27]. Also here the effects are per unit. For example, the *β* for age on joint damage is 1.034/year this means that for every year increase in age there is an increase of 3.4 % this is equal to an increase of 95.2 % every 20 years (1.034^20). All features (SJC, TJC, ACPA, RF, symptom duration, and age) were assessed at baseline

### Age and RA-related auto-antibodies

The presence of ACPA and RF were strong risk factors for radiographic progression (*β* = 1.37, *p* <0.001 and *β* = 1.30, *p* <0.001, respectively). These auto-antibodies can mediate the association between age and joint destruction if age is associated with a higher prevalence of these auto-antibodies. We observed no significant association between age at onset and presence of RF (odds ratio (OR) = 0.99, *p*= 0.09). The prevalence of ACPA was lower when RA was diagnosed at an older age (OR = 0.98, *p* <0.001). Therefore, these auto-antibodies did not mediate the association between age and joint damage (Table 2).

### Age and clinical measures of joint inflammation

Next we explored whether the extent of joint inflammation, measured using the SJC and TJC at baseline, was a mediator. Age at diagnosis was not associated with the number of joints involved (*β* = 1.00, *p* = 0.11 and *β* = 1.00, *p* = 0.55 for SJC and TJC, respectively). The SJC and TJC were also not associated with radiographic joint damage (*β* = 1.00, *p* = 0.23 and *β* = 1.00, *p* = 0.76); therefore these clinical measures of local inflammation were not mediators (Table 2).

### Age and serological measures of inflammation

Subsequently it was explored whether CRP, a measure of systemic inflammation, was a mediator. CRP-levels increased significantly with age (*β* = 1.016, *p* <0.001), indicating that with every year increase in age the CRP increased by 1.6 %. CRP-levels were also associated with severity of joint damage (*β* = 1.003, *p* = 0.003). In the third step of the mediation analysis, the association between age and joint damage was studied after adjustment for CRP. This revealed a very slight decrease in effect size, from 1.034 to 1.033 (Table 2), based on which it was concluded that also CRP was not a mediator. Similar results were seen when erythrocyte sedimentation rate (ESR) was studied (data not shown).

### Age and local inflammation measured by MRI

It is known that MRI of the extremities measures local inflammation more sensitively than physical examination and CRP [21, 22, 39]. To explore local inflammation in more detail, additional mediation analysis was performed in another group of patients with RA who underwent MRI of the extremities at baseline (Table 3). The MRI synovitis and bone marrow edema (BME) scores were summed, yielding the MRI inflammation scores. Also in this group of patients, an increase in age was associated with more joint damage at baseline; also here the effect was constant over time. (*β* = 1.032 *p* = 0.004). All subsequent analyses were adjusted for SJC and CRP to ensure that the results for MRI-detected inflammation were not explained by these traditional measures of inflammation. It was observed that patients presenting at older age had higher MRI inflammation scores (*β* = 1.018, *p* = 0.027) and that higher MRI inflammation scores were associated with radiographic joint damage at any point in time evaluated (*β* = 1.026, *p* = 0.018). Then in step 3 of the mediation analysis there was a decreased effect size of age with structural damage when additionally adjusting for the MRI inflammation score (*β* = 1.032 *p* = 0.004 to *β* = 1.025 *p* = 0.02), suggesting that MRI-detected inflammation is a partial mediator for the effect of age on radiographic joint damage.

**Table 3:** Mediation analysis in 56 patients with rheumatoid arthritis from the Leiden Early Arthritis Clinic, with radiographic severity of joint damage as the outcome

Step 1: effect of age on possible mediators			
	Effect (*β*)	95 % CI	*p* value
MRI inflammation	1.018	1.002–1.034	0.027
Synovitis	1.011	1.00–1.024	0.092
BME	1.021	1.00–1.043	0.052
Step 2: effect of age on radiographic joint damage			
	Effect (*β*)	95 % CI	*p* value
Ageing	1.032	1.010–1.055	0.004
Step 3: effect of age and possible mediator on radiographic joint damage			
	Effect (*β*)	95 % CI	*p* value
MRI inflammation	1.026	1.004–1.047	0.018
Ageing	1.025	1.004–1.047	0.021
Synovitis	1.069	0.97–1.17	0.15
Ageing	1.029	1.007–1.051	0.011
BME	1.039	1.011–1.067	0.007
Ageing	1.026	1.005–1.047	0.014

Step 1, 2 and 3 are explained in Fig. 1. In step 1 a linear regression is used, in step 2 and 3 a multivariate normal regression analysis is used [27]. The effects are per unit increase, for example per point increase in rheumatoid arthritis magnetic resonance imaging score (RAMRIS) and per year increase in age; for further explanation see legend of Table 2. *MRI* magnetic resonance imaging, *BME* bone marrow edema

## Discussion

The western population is ageing. Consequently the number of patients with RA diagnosed at an older age is rising [1, 2]. It is generally hoped that the additional years of life are spent in good health. RA, however, is associated with decreased functioning and quality of life. Previous studies on age and RA severity have contrasting results [6, 7, 9, 40, 41]. The present study of patients with RA, which included five longitudinal cohorts, showed that older patients had more severe joint damage at diagnosis and this effect remained present during the disease course. We evaluated several hypotheses to increase the understanding of the processes driving the observations of the influence of age. We observed that the effect was partially and modestly mediated by MRI-detected inflammation with increasing age.

Longitudinal data from five cohorts were studied on the effect of age on the severity of joint damage. Because of the presence of serial radiographic measurements multivariate normal regression analysis was used (this model is similar to a linear mixed model, only no random effect is added). For reasons of consistency this model was also used in the mediation analysis in the second part of the manuscript. However, the effect of joint damage was already present at baseline and the mediation analyses could also be done with joint damage at baseline as the outcome. Repeating the mediation analysis with baseline SHS as the outcome indeed revealed similar results (data not shown).

Interestingly, some variables assessed in the mediation analyses were inversely correlated with age. The symptom duration at the time of diagnosis was shorter at older age, indicating that older patients had less delay in getting access to rheumatologic care. Thus, although we hypothesized that older patients presented with more severe damage due to having longer duration of disease, older patients had a shorter period of symptoms at first presentation, hence arguing against this hypothesis. Also the prevalence of ACPA decreased at older age in 1,987-criteria-positive patients with RA, which is in contrast to the prevalence of ACPA in the general population [15], but a lower frequency of ACPA amongst older patients with RA has been described before [6]. Several studies have observed higher CRP-levels in older patients with RA [11, 16, 17], and we also observed this. However, the effect size of the association between age and severity of joint damage decreased very little after adjusting for CRP, therefore CRP was not considered an evident mediator.

MRI of the extremities is sensitive in detecting local inflammation and subclinical inflammation observed on MRI has been found to be relevant for radiographic progression of RA [21, 22]. The present data showed that patients with RA presenting at an older age have more MRI-detected inflammation. Ageing is associated with an increase in pro-inflammatory status and a decline in both T cell and B cell function [3–5]. Potentially, changes in the immune system are underlying the current observation of more severe inflammation on MRI at an older age. Earlier studies have shown that the severity of MRI-detected inflammation is associated with the severity of radiographic joint damage and that this effect is independent of the effects of CRP and SJC on radiographic progression [22, 39]. The present data also show that MRI-detected inflammation is associated with severity of joint damage, independent of other measures of inflammation. Hence in the last two steps of the mediation analysis, adjustments for CRP and SJC were made. The finding that the effect size of age decreased after additional adjustment for MRI-detected inflammation suggests that the effect of more severe joint damage at higher age is partly mediated by the presence of more severe MRI-detected inflammation at an older age. In other words, it suggests that MRI-inflammation acts in the so-called causal path [36].

Interestingly, some recent evidence suggested that older age might also be associated with presence of more MRI-detected inflammation in symptom-free persons [42]. More studies are needed to determine the validity of these results, and to differentiate disease-related inflammation on MRI from variations that are present in the general population. Nonetheless, in our view this does not affect the validity of the present results. If MRI-detected inflammation also occurs at an older age in people without RA, this most likely does not affect the mediation analyses in RA and does not influence the decrease in the beta value for age between steps 2 and 3.

We explored whether the higher SHS scores at older age were due to age-related degeneration. We have tried to detangle these effects by evaluating JSN scores and erosion scores separately and predilection locations for degenerative changes separately. Although these comparisons do not allow us to make conclusions about the causality, degenerative changes at an older age appear insufficient to explain the observed association between age and severity of joint damage.

Similarly, it can be questioned whether more severe MRI-detected inflammation is RA-specific or age-specific. This is even more difficult to discriminate as the RAMRIS was derived for RA; degenerative features such as JSN and osteophytes were not included; also the locations assessed by RAMRIS are specific for RA. The CMC-1 joint is included but is known to be affected by degeneration as well. When we evaluated the BME scores in the base of metacarpal-1 and trapezium (the two bones together forming CMC-1), the correlation with age was not significant (*ρ* = 0.24, *p* = 0.07) whilst the BME score obtained in other bones in the wrist was positively correlated with age (*ρ* = 0.41, *p* = 0.002). This suggests that the higher MRI scores seen in patients with RA at higher age were not primarily due to degeneration (in the process of osteoarthritis).

Degeneration in the light of osteoarthritis may be different from the more global effects of wear and tear. We cannot exclude that part of the effect of greater MRI-detected inflammation at an older age is due to wear and tear. Part of the observation of more radiographic erosions at older ages can, in the same path, also be due to wear and tear. If this is true, hand and foot radiographs of healthy people at older ages would also show erosions according to the SHS method. To the best of our knowledge, radiographic studies on the hands and feet of healthy persons of different age categories have not been done.

It has been suggested that older patients are treated differently in comparison to younger patients [43–46], but others have argued against this [47]. The majority of patients studied here were included in periods when early, tailored, treatment and use of biologic agents were uncommon. Importantly, treatment most likely does not affect the results of our study as differences in radiographic joint damage were already present at baseline. The differences were already present before the start of treatment, so treatment was not a likely mediator.

Analyses were adjusted for gender to account for differences in male/female ratios at different ages. Additionally, women have hormonal changes during their lifetime. When repeating the mediation analyses in men only, no differences were observed (data not shown), suggesting that gender was not a confounder.

The strength of this study is that five cohorts with longitudinal data were studied. The cohorts used different inclusion criteria, but despite these differences, all cohorts had more radiographic joint damage with older age at disease onset. This replication supported the validity of the association between age and severity of joint damage. A limitation is that the mediation analyses were performed using data from one cohort only. However, data on the complete set of potential mediators were not available for the other datasets. Another limitation is the relatively small number of patients with RA with baseline MRI data and 1-year follow up in relation to the large cohorts of patients with radiographic data. This is due to the fact that in our setting MRI was not available until a few years ago. Notably, the effect size of age on radiographic joint damage in this small patient group of patients with RA was almost similar to that of the larger RA datasets.

## Conclusions

The present study convincingly showed that patients with RA diagnosed at an older age already have more joint damage on disease presentation, and this effect remains during the disease course. This effect might be partially explained by more severe local inflammation at an older age. Future studies are needed to elucidate the biological mechanisms determining inflammation severity and RA severity and changes during the patient’s lifetime.

## Additional files

Additional file 1: **Supplementary material.** (DOC 33 kb)

Additional file 2: Figure S1. Residuals of the multivariate normal regression models of five included cohorts. (TIFF 114 kb)

Additional file 3: Figure S2. The severity of joint damage in group of patients with different age categories. (TIFF 114 kb)

Additional file 4: Figure S3. Correlation between age and the total Sharp-van der Heijde score at different locations (B). (TIFF 102 kb)

## Abbreviations

ACPA: anti-citrullinated-peptide-antibodies
BME: bone marrow edema
CMC: carpometacarpal
CRP: C-reactive-protein
DMARD: disease-modifying antirheumatic drug
EAC: Early Arthritis Clinic
ICC: intra-reader intra-class correlation coefficient
JSN: joint space narrowing
MCP: metacarpophalangeal
MRI: magnetic resonance imaging
MTP: metatarsophalangeal
PIP: proximal interphalangeal
RA: rheumatoid arthritis
RAMRIS: rheumatoid arthritis magnetic resonance imaging score
RF: rheumatoid factor
SHS: Sharp-van der Heijde
TJC: tender joint count
SJC: swollen joint count
