# Supplementary material for: Studies on ageing and the severity of radiographic joint damage in rheumatoid arthritis
Source: Arthritis Res Ther. 2015 Aug 24;17(1):222. doi: 10.1186/s13075-015-0740-0 (PMC4547419; doi:10.1186/s13075-015-0740-0)
Supplement: Supplementary file 1 — Supplementary material. (DOC 33 kb) [file 13075_2015_740_MOESM1_ESM.doc]

**Additional file 1**

**Supplementary methods: MRI scan protocol**

MR imaging of the hand (wrist and metacarpophalangeal joints) and forefoot (metatarsophalangeal joints) was performed within two weeks after inclusion, at the most painful side, or in case of completely symmetric symptoms at the dominant side. The presence of clinical arthritis at physical examination of the joints that were scanned was not a prerequisite. Patients with impaired renal function or known hypersensitivity or allergic reactions to contrast media were imaged without contrast administration (n=2). MR imaging was performed on a MSK-extreme 1.5T extremity MR imaging system (GE, Wisconsin, USA) using a 145mm coil for the foot and a 100mm coil for the hand. The patient was positioned in a chair beside the scanner, with the hand or foot fixed in the coil with cushions. The forefoot was scanned using a T1-weighted fast spin-echo (FSE) sequence in the axial plane with repetition time (TR) of 650 ms, echo time (TE) 17ms, acquisition matrix, 388×288, echo train length (ETL) 2; and a T2-weighted FSE sequence with frequency selective fat saturation in the axial plane (TR/TE 3000/61.8; acquisition matrix 300x224, ETL7). Due to time constraints, imaging of the foot was limited to pre-contrast sequences only. In the hand, the following sequences were acquired before contrast injection: T1-weighted FSE sequence in the coronal plane (TR/TE 650/17ms; acquisition matrix 388×88; ETL2); T2-weighted FSE sequence with frequency selective fat saturation in the coronal plane (TR/TE 3000/61.8ms; acquisition matrix, 300x224, ETL7). After intravenous injection of gadolinium contrast (gadoteric acid, Guerbet, Paris, France, standard dose of 0.1 mmol/kg) the following sequences were obtained: T1-weighted FSE sequence with frequency selective fat saturation in the coronal plane (TR/TE 650/17ms, acquisition matrix 364×224, ETL2), T1-weighted FSE sequence with frequency selective fat saturation in the axial plane (TR/TE 570/7 ms; acquisition matrix 320x192; ETL2). Field-of-view was 100mm for the hand and 140mm for the foot. Coronal sequences had 18 slices with a slice thickness of 2mm and a slice gap of 0.2mm. All axial sequences had a slice thickness of 3mm and a slice gap of 0.3mm, with 20 slices for the hand and 16 for the foot. Total imaging time was approximately 75 minutes.

The total MRI-inflammation scores is the sum of the RAMRIS scores for synovitis and bone marrow edema.
